# Cost-Utility Analysis of Once-Weekly Semaglutide, Dulaglutide, and Exenatide for Type 2 Diabetes Patients Receiving Metformin-Based Background Therapy in China

**DOI:** 10.3389/fphar.2022.831364

**Published:** 2022-02-18

**Authors:** Shanshan Hu, Shuowen Wang, Chendong Qi, Shengying Gu, Chenyang Shi, Lin Mao, Guorong Fan

**Affiliations:** Department of Clinical Pharmacy, Shanghai General Hospital, Shanghai Jiao Tong University School of Medicine, Shanghai, China

**Keywords:** subcutaneous semaglutide, dulaglutide, extended-release exenatide, type 2 diabetes, UKPDS OM, cost-utility analysis, once-weekly GLP-1 receptor agonist

## Abstract

**Introduction:** The substantial financial burden associated with type 2 diabetes (T2D) over a lifetime cannot be neglected. Therefore, the objective of this study was to evaluate the pharmacoeconomic value of three once-weekly GLP-1 RAs, namely subcutaneous semaglutide (sc. SEMA), dulaglutide (DULA), and extended-release exenatide (e-r EXEN), in treating patients with T2D that cannot be controlled with metformin-based background therapy, and to find a suitable price reduction for non-cost-effective medications, to provide reasonable recommendations to the administration for adjusting drug prices.

**Methods:** The baseline characteristics of the simulation patient cohort were sourced from a comprehensive meta-analysis synthesizing 453 trials evaluating 21 hypoglycemic agents from nine categories of drugs. The UKPDS OM2 was applied to project the long-term effectiveness and costs from a Chinese health care provider’s perspective. After cost-utility analysis, the reasonable price adjustment of non-cost-effective options was explored via binary search. Uncertainty was measured by means of sensitivity analysis.

**Results:** After a 40-year simulation, the sc. SEMA, DULA, and e-r EXEN groups yielded 9.6315, 9.5968, and 9.5895 quality-adjusted life years (QALYs), respectively. In terms of expenditure, the total costs for the sc. SEMA, DULA, and e-r EXEN groups were $42012.47, $24931.27, and $40264.80, respectively. DULA was dominant over e-r EXEN due to the higher QALYs and lower total costs. The ICURs of sc. SEMA vs. DULA and sc. SEMA vs. e-r EXEN were $492994.72/QALY and $41622.69/QALY (ICUR > λ), respectively, indicating that sc. SEMA was not more cost-effective than DULA or e-r EXEN. The INMB and absolute NMB yielded the same conclusions which were robust to one-way, scenario, and probabilistic sensitivity analyses. After several assumptions in the binary search, sc. SEMA and e-r EXEN appear to become cost-effective when their annual costs are decreased by 57.67% and 70.34%, respectively, with DULA as a counterpart.

**Conclusion:** From the cost-utility analysis, DULA appears to be the most cost-effective option among sc. SEMA, DULA, and e-r EXEN for the treatment of patients with T2D receiving metformin-based background therapy. With a 57.67% or 70.34% reduction in cost, sc. SEMA or e-r EXEN, respectively, would become as cost-effective as DULA in China.

## 1 Introduction

The current incidence and prevalence of diabetes are rapidly increasing worldwide as a result of population aging, urbanization, and associated lifestyle changes. The worldwide diabetes map, delivered by the International Diabetes Federation (IDF), showed that 463 million individuals (9.3%) had diabetes in 2019, and this number is projected to reach 578 million (10.2%) by 2030 and 700 million (10.9%) by 2045 (Saeedi et al., 2019). As indicated by IDF information, 66% of individuals with diabetes live in metropolitan regions, and three out of four are of working age. More than 4,000,000 adults died from diabetes-related causes in 2019. In China, diabetes has become a serious public health problem due to high mortality and socioeconomic burden. The age-standardized death rate per 100,000 people among diabetic patients increased by 2.0% from 1990 to 2013 (Zhou et al., 2016). Among adult diabetic patients in China, there is a close connection between diabetes and increased mortality due to a wide range of cardiovascular and non-cardiovascular diseases (Bragg et al., 2017). Type 2 diabetes (T2D) accounts for the vast majority (approximately 90%) of diabetes (Chen et al., 2011). Notably, T2D has become more common in adolescents, and unfavorable metabolic control among youth with the disease will substantially influence the future weight of the T2D burden (Pinhas-Hamiel and Zeitler, 2005; Lawrence et al., 2009).

The foundation of T2D treatment consists of sufficient diabetes education and lifestyle modifications (Teo et al., 2020). Early control of glycemia in diabetic patients can effectively reduce the occurrence of complications and potentially slow the progression of diabetes (Jia et al., 2019). A combination of medicine regimens with different pathophysiological pathways could achieve higher health outcomes. According to the guidelines (Jia et al., 2019), the current hypoglycemic drugs can be classified into new classes of hypoglycemic agents such as dipeptidyl peptidase-4 inhibitors (DPP-4i), sodium–glucose cotransporter-2 inhibitors (SGLT-2i), and glucagon-like peptide-1 receptor agonists (GLP-1 RAs), traditional hypoglycemic agents such as metformin, pioglitazone, sulfonylureas, and α-glucosidase inhibitors, and insulins. GLP-1 RAs have been recommended as first-line therapy for T2D patients at high cardiovascular risk. Moreover, GLP-1 RAs have been found to have multifactorial advantages, such as reduction in body weight, decreased risk of cardiovascular death, and renal protection (Htike et al., 2017; Garber et al., 2020; Nauck et al., 2021).

Since the first GLP-1 RA was successfully approved by the US Food and Drug Administration (FDA) in 2005, following intensive studies and the gradual accumulation of evidence, the status of this kind of agent in the treatment of T2D has been continuously improving. An increasing number of countries have included GLP-1 RAs as a primary therapy for T2D treatment (Buse et al., 2020). The currently marketed GLP-1 RAs include exenatide, lixisenatide, liraglutide, dulaglutide, albiglutide, and semaglutide (Müller et al., 2018). Among the GLP-1 RAs, semaglutide is the only one available as an oral preparation, but it has not been marketed yet in China. Other GLP-1 RAs can be categorized as short-acting and long-acting treatments (Fineman et al., 2012; Albèr et al., 2017). At present, three long-acting GLP-1 RAs, such as subcutaneous semaglutide (sc. SEMA), dulaglutide (DULA), and extended-release exenatide (e-r EXEN), have been on the market in China and are now used in the clinic (Alatorre et al., 2017; Gentilella et al., 2019).

Compared to EXEN twice daily, e-r EXEN has a moderately steady plasma concentration because the extended-release system provides continuous delivery of the medicine leading to an equal or slightly improved clinical effect on glycemic control and body weight (Wysham et al., 2018). sc. SEMA once weekly has been approved for T2D patients by the FDA and has been marketed since 2017. The half-life of sc. SEMA was extended to 160 h by chemical modifications, which supported once-weekly administration (Lau et al., 2015; Doggrell, 2018). Sc. SEMA can attain a body weight reduction up to 7% and HbA1c reduction by 1.8% over 40 weeks of treatment and can reduce the risk for adverse cardiovascular events for T2D patients with high cardiovascular risk (Marso et al., 2016; Nauck et al., 2016; Zweck et al., 2019). DULA entered the Chinese market in 2019 and was added to the drug list of the national medical insurance in 2020; it is the only currently available once-weekly GLP-1 RA in the national health care security system. DULA showed a nearly 3% body weight reduction and 1.4% hemoglobin A1C (HbA1c) reduction after a 26-week trial (Dungan et al., 2014).

Currently, few studies have aimed to estimate the long-term cost-effectiveness of once-weekly GLP-1 RAs for T2D patients receiving metformin-based background therapy in China. However, the formidable financial burden associated with T2D over the lifetime cannot be neglected. Therefore, the aim of this study was to evaluate the pharmacoeconomic value of three once-weekly GLP-1 RAs, namely sc. SEMA, DULA, and e-r EXEN in treating patients with T2D that cannot be controlled with metformin-based background therapy. After pharmacoeconomic estimation, a binary search was applied to identify a suitable price reduction for non-cost-effective medications to give policy makers a reasonable recommendation for adjusting the drug price.

## 2 Methods

### 2.1 UKPDS Outcomes Model (version 2)

The United Kingdom Prospective Diabetes Study Outcomes Model (version 2) (UKPDS OM2) is an individual-level computerized simulation tool for evaluating long-term health outcomes and expenditures, whose algorithms were derived from the UKPDS 82 trial (Hayes et al., 2013). The UKPDS OM2 has been previously released and validated (Pollock et al., 2019). The UKPDS OM2 forecasts the disease progression of diabetic complications, including congestive heart failure (CHF), ischemic heart disease (IHD), myocardial infarction (MI), stroke, amputation, renal failure, blindness, and diabetic ulcers (University of Oxford, D.T.U., Health Economics Research Centre, 2015). The detailed model structure and algorithms can be found in our previous papers and Supplementary Figure S1 [see additional file] (Hu et al., 2021a; Hu et al., 2021b). The baseline characteristics, high-risk factors, history events, costs, health utility, and other parameters were input into the model. The life expectancy (LE), quality-adjusted life years (QALYs), and total costs of type 2 diabetes patients were reported from the model. In this study, a 40-year simulation was used to capture the whole disease progression of diabetic complications throughout a patient’s lifetime with default annual cycles. The discount rate for health utilities and costs was 5% (Hou et al., 2019) in keeping with the World Health Organization (WHO) guidelines (World Health Organization, 2002). Furthermore, Monte Carlo simulations were performed to solve parameter uncertainties.

### 2.2 Clinical Efficacy Data and Simulation Cohort

The comparative efficacy data of sc. SEMA, DULA, and e-r EXEN for T2D patients receiving metformin-based background therapy were sourced from a newly published network meta-analysis (Tsapas et al., 2020). The meta-analysis synthesized 453 trials evaluating 21 hypoglycemic agents from nine categories of drugs (Tsapas et al., 2020). In our study, the clinical efficacy data of sc. SEMA, DULA, and e-r EXEN were extracted from the comparators of each medication versus placebo (Table 1) in the subgroup of patients receiving metformin-based background therapy. Each group assumed 1000 simulated patients. The baseline characteristics of the patient cohort are shown in Table 2 in detail. In the simulation cohort, the mean age, median duration of diabetes, proportion of females, and mean HbA1c were 60.62 years, 9.99 years, 40.76%, and 8.07%, respectively. Other data on baseline characteristics not reported in the meta-analysis, such as the proportion of race, medical history, and smoking status, were extracted from a study that focused on 15,252 T2D patients with an average follow-up time of 8.2 years.

**TABLE 1:** Change in the HbA1c level in patients receiving metformin-based background therapy.

Group vs. Placebo	Mean difference (MD)	95% CI	
		Lower	Upper
Subcutaneous semaglutide	−1.33	−1.50	−1.16
Dulaglutide	−0.89	−1.05	−0.73
Extended-release exenatide	−0.80	−0.99	−0.62

Note: HbA1c: hemoglobin A1c; 95% CI: 95% confidence interval.

**TABLE 2:** Baseline characteristics of the simulation cohort.

Trial characteristic	Mean	SD (or range)
Total simulation sample	1000	
Mean age, years	60.62	
Female, %	40.76	
Race, %^a^		
White	62.70%	
Black/African American	18.00%	
Other^b^	19.30%	
Median duration of diabetes, years	9.99	
Mean HbA1c, %	8.2	8.0–8.5
BMI	31.2	
Mean body weight, kg	87.63	9.17
Height, meters	1.68	
History of MI^a^	12.50%	
History of angina^a^	7.60%	
Smoking status, %^a^	10.70%	

Note:

HbA1c: hemoglobin A1c; BMI: body mass index; MI: myocardial infarction; SD: standard deviation.

Data source: Tsapas et al. (2020), Neuwahl et al. (2021).

a Other data not reported in the meta-analysis were extracted from Neuwahl et al. (2021).

b Includes patients whose race was not available in study records.

### 2.3 Costs and Utilities

Direct medical costs were collected and inflated to 2020 Chinese yuan (¥) with the consumer price index (CPI) and expressed in 2020 US dollar ($) ($1 = ¥6.8974, 2020), including medication costs, T2D-associated complication costs, and T2D management costs. The unit of medication costs of sc. SEMA, DULA, and e-r EXEN were based on the average official government bid price in China in 2020 (Table 3). T2D management costs were sourced from a prospective cohort study in China (Li et al., 2019). T2D-related complication costs comprised fatal, nonfatal, and maintenance costs and were collected from the literature (Shao et al., 2017; Cai et al., 2019; Hou et al., 2019). After 5 years, all the patients were switched to insulin glargine U100, as T2D progressed with deterioration of the beta-cell function (Gao et al., 2012).

**TABLE 3:** Costs of medications.

Medication	Unit of cost (¥/box)	Specification	Usual dosage	Annual cost ($)	Lower limit (−20%) ($)	Upper limit (−20%) ($)
DULA	298.00	1.5 mg: 0.5 ml/piece, two pieces/box	1.5 mg qw.	1036.91	829.53	1244.29
e-r EXEN	2068.86	2 mg^a^4/box, each box contains four single-dose kits	2 mg qw.	3599.38	2879.50	4319.25
sc. SEMA	1120.00	1.34 mg/ml, 1.5 ml, one piece/box	1 mg qw.	3491.17^a^, 3897.12^a^	3117.70^b^	4676.54^b^

Note:

DULA: dulaglutide; e-r EXEN: extended-release exenatide; sc. SEMA: subcutaneous semaglutide; qw: once a week.

Lower limit (-20%): the lower limit of the annual cost was defined as 20% down of the annual cost; Upper limit (+20%): the upper limit of the annual cost was defined as 20% up of the annual cost.

1$ = ¥6.8974, 2020.

a sc. SEMA treatment followed a fixed dose-escalation regimen: 0.25 mg for 4 weeks, then 0.5 mg for 4 weeks, and then a maintenance dose of 1.0 mg. Therefore, the medication cost of sc. SEMA for the first year is $3491.17, and the maintenance annual cost is $3897.12.

b Data were calculated from the maintenance annual cost of sc. SEMA.

Health state utility scores are a numerical representation of health preference and range from 0 to 1. At the extremes, a utility score of 0 implies death, and a utility score of 1 implies perfect health. The initial utility of T2D patients without complications was deemed to be 0.876 (Pan et al., 2016; Hou et al., 2019). The quality of health state utility and disutility scores were taken from a 5-level, 5-dimensional EuroQol scale (EQ–5D–5L) study of Chinese T2D patients (Pan et al., 2016; Hou et al., 2019). Other essential values not reported in the survey were derived from the UKPDS 62 study (Clarke et al., 2002) and other utility-related studies (Wu et al., 2018a; Wu et al., 2018b; Cai et al., 2019). All the costs and utilities input into the UKPDS OM2 are shown in Table 4 in detail.

**TABLE 4:** Key model inputs of costs and utilities.

Complication	At the time of event			In subsequent years	
	Fatal cost	Nonfatal cost	Utility decrement	Cost	Utility decrement
IHD	0.00	6,451.66	−0.090	1,151.78	−0.090
MI	8,052.81	8,052.81	−0.055	496.72	−0.236
Heart failure	3,110.07	3,110.07	−0.236	1,644.48	−0.236
Stroke	2,323.34	3,136.05	−0.164	552.89	−0.326
Amputation	4,546.19	4,546.19	−0.380	4,425.28	−0.380
Blindness	—	2,420.92	−0.157	1,790.97	−0.157
Renal failure	0.00	15,055.46	−0.400	15,055.46	−0.400
Ulcer	—	2,368.13	−0.059	833.47	−0.059
Initial utility	0.876				
Cost in the absence of complications	1,463.01				

Note:

IHD: ischemic heart disease; MI: myocardial infarction.

Costs were collected from published literatures on Chinese economic evaluation and expressed in 2020 US dollars.

### 2.4 Cost-Utility Analysis

The cost-utility analysis was measured by two main outcomes: the incremental cost-utility ratio (ICUR) and the incremental net monetary benefit (INMB). Both the ICUR and INMB are calculated from three elements: incremental effectiveness (ΔQALY), incremental total costs (ΔCost), and willingness-to-pay thresholds. The QALYs and total costs were simulated and output from the UKPDS OM2. The willingness-to-pay thresholds have been recommended for developing countries, at 1- to 3-fold gross domestic product (GDP) per capita, by the WHO (Bertram et al., 2016). In China, the value of GDP per capita is $10503.52 (equal to ¥72447), and apparently, the value of 3-fold GDP per capita is $31510.57 (equal to ¥217341) in 2020. In this study, the cost-effective threshold was set at $31510.57/QALY, the 3-fold GDP per capita value, and defined as λ.

The formula of the ICUR and decision rules is as follows:

ICUR=ΔCost/ΔQALY

Decision rules:

Assume group A vs. B:
** **If ΔCost <0 and ΔQALY >0:
**  **Then, group A is implied to be dominant;
** **If ΔCost >0 and ΔQALY <0:
**  **Then, group B is implied to be dominant;
** **If ΔCost >0 and ΔQALY >0:
**  **Then, ask for ICUR:
**   **If ICUR > λ:
**    **Then, group A is implied not to be more cost-effective than group B;
**   **If ICUR < λ:
**    **Then, group A is implied to be more cost-effective than group B.

The formula of the NMB and decision rules is as follows:

① INMB:

INMB=ΔQALY∗λ-ΔCost

Decision rules:

Assume group A vs. B:
** **If INMB >0:
**  **Then, group A appears to be more cost-effective than group B;
** **If INMB <0:
**  **Then, group A appears not to be more cost-effective than group B.
② Absolute NMB:

Absolute NMB=QALY∗λ-Cost

Decision rules:

Assuming group A vs. B vs. C, then the rank order for absolute NMB represents the rank order for the cost-effectiveness of groups A, B, and C:
If absolute NMB_A_ > absolute NMB_B_ > absolute NMB_C_:
Group A appears to be the most cost-effective strategy among groups A, B, and C, followed by group B and then group C.

### 2.5 Sensitivity Analysis

Sensitivity analyses, such as one-way sensitivity analysis (1-w SA) and probabilistic sensitivity analysis (PSA), were performed to measure the impact of uncertainty around input parameters, as long-term outcomes were potentially derived from short-term data with concern.

Costs, utilities, and other relative parameters were captured in the 1-w SA with details shown in Table 5. More concisely, the treatment time for patients who received sc. SEMA, DULA, and e-r EXEN was assumed to be 4 and 6 years. The time horizon was simulated from 30 to 50 years. The initial utility of T2D patients was set at 0.78 and 0.92. T2D-related complication costs and disutilities were varied from the lower limit to the upper limit of their 95% CIs, with the discount rate varying from 3% to 8%. T2D-related complication costs and disutilities were alternatively varied by ±20% and ±10%, respectively, when the 95% CIs data could not be acquired. The results of the 1-w SA were illustrated by tornado diagrams.

**TABLE 5:** Parameters for sensitivity analysis.

No.	Parameter	Baseline	Low	High
1	Discount rate	5%	3%	8%
2	Initial utility	0.876	0.78	0.92
3	Treatment time, years	5	4	6
4	Time horizon, years	40	30	50
Cost, $				
5	IHD per year cost (±20%)	1151.78	921.43	1382.14
6	MI per year cost	496.71	314.79	678.65
7	CHF per year cost	1644.49	1368.42	2871.11
8	Stroke per year cost	552.89	486.36	903.12
9	Blindness per year cost	1790.97	1560.17	2021.65
10	ERSD per year cost	15055.46	14347.15	15890.96
11	Amputation per year cost	4425.26	0.00	7862.31
12	Ulcer per year cost (±20%)	833.47	666.77	1000.16
Health disutility scores				
13	IHD disutility scores (±10%)	0.09	0.081	0.099
14	MI disutility scores	0.236	0.026	0.446
15	CHF disutility scores	0.236	0.026	0.446
16	Stroke disutility scores	0.326	0.036	0.616
17	Blindness disutility scores	0.157	0.007	0.307
18	ERSD disutility scores	0.4	0.19	0.61
19	Amputation disutility scores	0.38	0.204	0.496
20	Ulcer disutility scores(±10%)	0.059	0.0531	0.0649

Note:

IHD, ischemic heart disease; MI, myocardial infarction; ERSD, end-stage renal disease.

The range data of IHD per year cost, ulcer per year cost, IHD disutility score, and ulcer disutility score were not reported. Therefore, we tested IHD and ulcer per year costs as ±20% and IHD and ulcer utility score as ±10%.

PSA, for measuring the second-order uncertainty, was sampled around model inputs with a fixed probability distribution by the Monte Carlo approach over 1,000 iterations. The PSA results were depicted by scatter plots.

### 2.6 Binary Search

The binary search always starts from the middle of the specific sequence to search for the target value, which can save half of the search time (Hu et al., 2021b). In this study, a binary search was applied to explore the suitable price reduction for non-cost-effective medication in the cost-utility analysis.

### 2.7 Assumptions

There were some assumptions during the research. First, the baseline simulation cohort was consistent with that reported in the subgroup of patients receiving metformin-based background therapy of the Tsapas’ article (Tsapas et al., 2020) in order to simulate the clinical practice. Second, the diabetes management costs in three interventions were assumed as same, due to the comparable safety profile of the three GLP-1 RAs (Ahmann et al., 2018; Pratley et al., 2018). Third, the transition probabilities, distributions, and cycle length were defaulted by UKPDS OM2 using equations from the UKPDS 82 trial.

## 3 Results

### 3.1 Base-Case Results

After a 40-year simulation, the long-term health outcomes and expenditures of sc. SEMA, DULA, or e-r EXEN in treating T2D patients receiving metformin-based background therapy are listed in Table 6. For benefits, the sc. SEMA, DULA, and e-r EXEN groups yielded 9.6315, 9.5968, and 9.5895 QALYs, respectively. For expenditures, direct medication treatment for the sc. SEMA, DULA, and e-r EXEN groups costs $23330.28, $6248.60, and $21597.42, respectively; the T2D-related complication costs were $18682.19, $18682.67, and $18667.38, respectively; and total costs were $42012.47, $24931.27, and $40264.80, respectively.

**TABLE 6:** Long-term outcomes in the base-case cost-utility analysis.

Group	sc. SEMA	DULA	e-r EXEN	sc. SEMA vs. DULA	sc. SEMA vs. e-r EXEN	DULA vs. e-r EXEN
LE, years	11.6320	11.6058	11.5919	0.0262	0.0401	0.0139
LE, [95% CIs]	11.4870–11.9072	11.4496–11.8771	11.4513–11.8756	—	—	—
QALYs, QALY	9.6315	9.5968	9.5895	0.0346	0.0420	0.0073
QALYs [95% CIs]	9.5213–9.8542	9.4796–9.8225	9.4710–9.8189	—	—	—
Therapy costs ($)	23330.28	6248.60	21597.42	17081.68	1732.86	−15348.82
Cost of complications ($)	18682.19	18682.67	18667.38	−0.48	14.81	15.29
Total cost ($)	42012.47	24931.27	40264.80	17081.20	1747.67	−15333.53
ICUR (QALY)	—	—	—	492994.72	41622.69	Dominance
INMB ($)	—	—	—	−15989.43	−424.59	15564.83
Absolute NMB ($)	261480.43	277469.86	261905.03	—	—	—

Note:

LE, life expectancy; CIs, confidence intervals; QALYs, quality-adjusted life years; ICUR, incremental cost-utility ratio; INMB, incremental net monetary benefit; sc. SEMA, subcutaneous semaglutide; DULA, dulaglutide; e-r EXEN, extended-release exenatide; vs., versus.

ICUR = ΔCost/ΔQALY.

INMB = ΔQALY*λ—ΔCost.

Absolutely NMB = QALY*λ - Cost.

Willingness-to-pay thresholds (λ): $31510.57.

Overall, the ICUR of sc. SEMA vs. DULA was $492994.72/QALY, which surpassed $31510.57/QALY (λ), indicating that sc. SEMA was not more cost-effective than DULA. Likewise, the ICUR of sc. SEMA vs. e-r EXEN was $41622.69/QALY (ICUR > λ), revealing that sc. SEMA was not more cost-effective than e-r EXEN either. Nevertheless, DULA was dominant to e-r EXEN due to the higher QALYs and lower costs. The INMB indicator demonstrated the same results. The ranking of absolute NMB was DULA, e-r EXEN, and sc. SEMA, showing that DULA was the dominant strategy among the three.

### 3.2 Sensitivity Results

**FIGURE 1:**
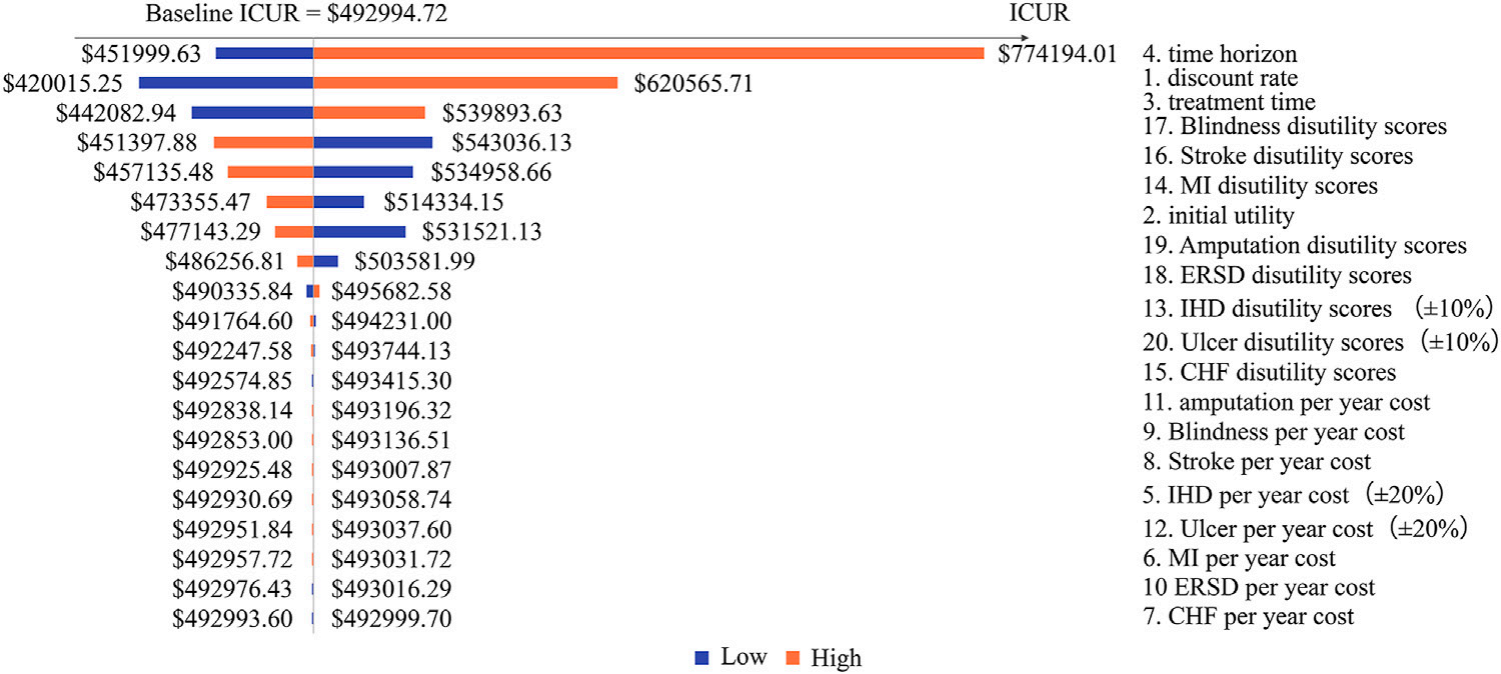
Tornado diagram of the one-way sensitivity analysis (SEMA group vs. DULA group).

**FIGURE 2:**
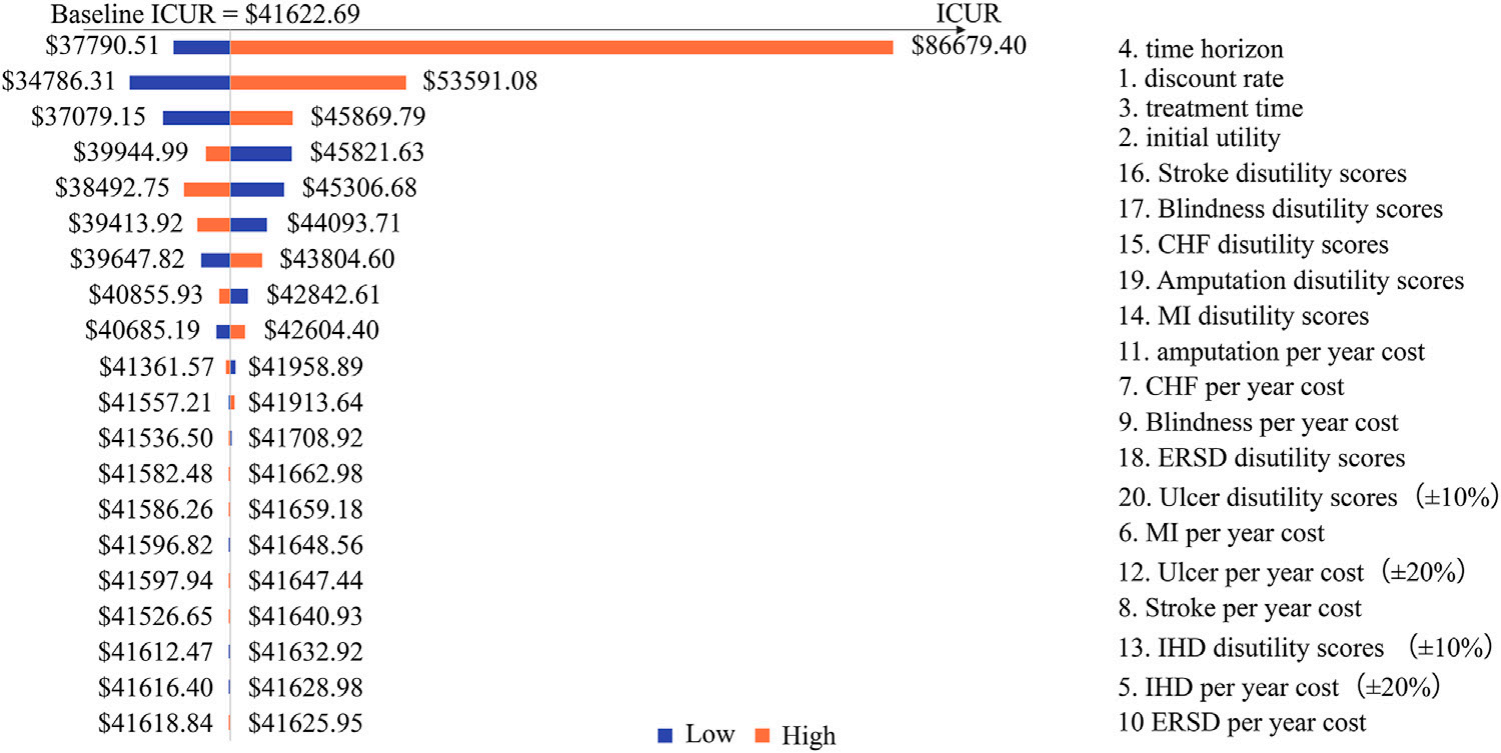
Tornado diagram of the one-way sensitivity analysis (SEMA group vs. e-r EXEN group).

Across all the 1-w SAs and PSAs, DULA remained dominant among the three strategies. A total of 20 potential factors associated with model inputs and assumptions were included in the 1-w SA, among which the time horizon and the discount rate had a comparatively large impact on the outputs. The tornado diagram of DULA vs. e-r EXEN was not depicted because DULA was always dominant regardless of the model inputs. The tornado diagrams of sc. SEMA vs. DULA and sc. SEMA vs. e-r EXEN are shown in Figures 1, 2.

Time horizon has a relatively large influence on the model outputs from tornado diagrams. Therefore, a scenario analysis for 10, 20, 30, 40 years (base case), and 50 years of time horizon was applied to improve the reliability of the conclusion (Table 7), further confirming the conclusion.

**TABLE 7:** Results of scenario analysis on time horizon.

ICUR, $/QALY	Base case	Simulation of cost-effectiveness at five time horizons, $/QALY				
		10 years	20 years	30 years	40 years	50 years
sc. SEMA vs. DULA	492994.72	1536812.27	737032.98	451999.63	492994.72	774194.01
sc. SEMA vs. e-r EXEN	41622.69	266507.96	48864.09	37790.51	41622.69	86679.40
DULA vs. e-r EXEN	Dominance	Dominance	Dominance	Dominance	Dominance	Dominance

Note:

Dominance: DULA is dominant with higher QALYs and lower costs than e-r EXEN.

**FIGURE 3:**
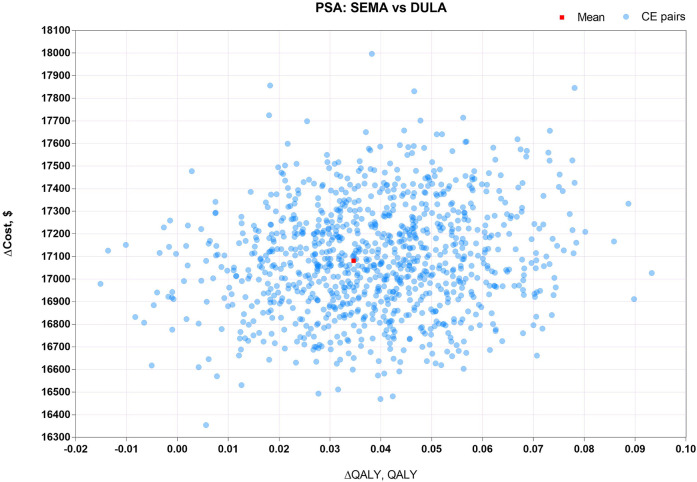
Scatter plots of ICUR for sc. SEMA vs. DULA.

**FIGURE 4:**
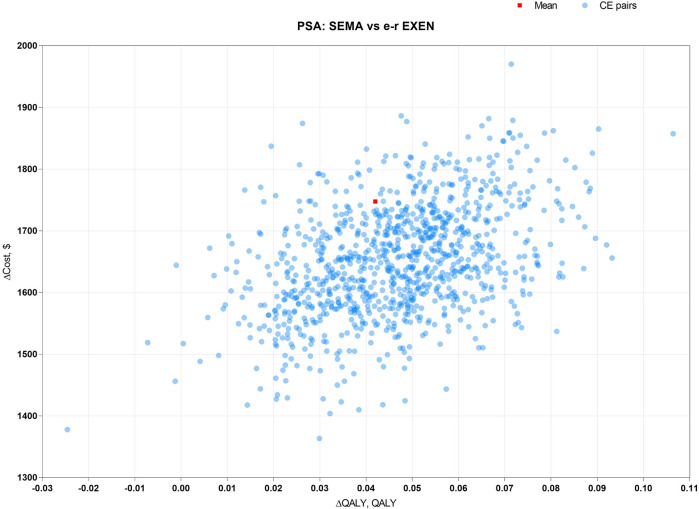
Scatter plots of ICUR for sc. SEMA vs. e-r EXEN.

**FIGURE 5:**
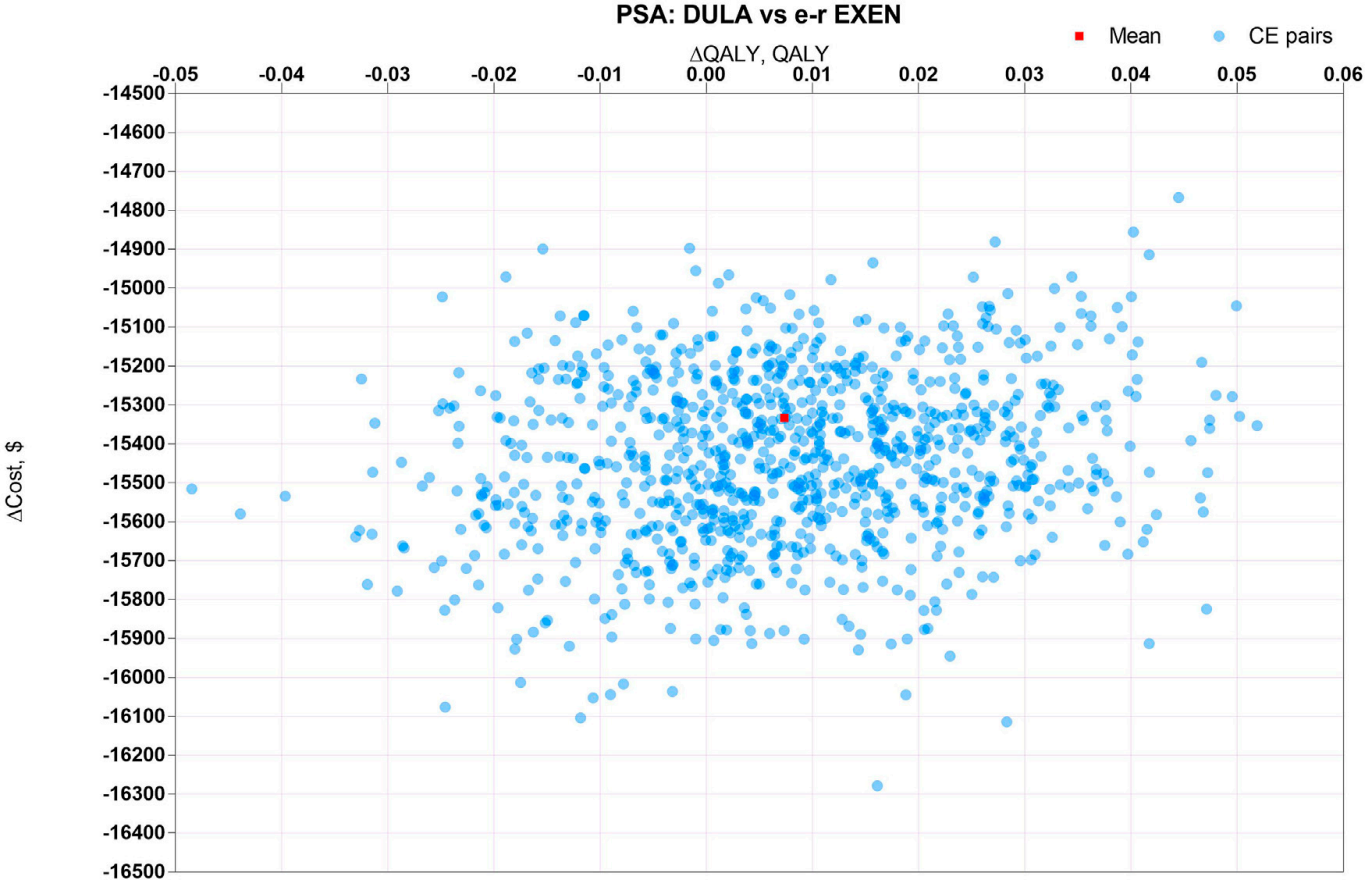
Scatter plots of ICUR for sc. DULA vs. e-r EXEN.

The results of PSA, with 1000 cohort patients over 1000 iterations of the Monte Carlo simulation, are depicted by scatter plots of ICUR in Figures 3–5. Figure 3 shows that there was a 100% probability that sc. SEMA was not more cost-effective than DULA, as the line of y = λx is far below the scatter point of CE pairs. There was a 1.8% probability that DULA was dominant to sc. SEMA, as it had a higher QALY and lower cost. Figure 4 illustrates that there was a 100% probability that sc. SEMA was not more cost-effective than e-r EXEN, and there was a 0.4% probability that e-r EXEN was dominant to sc. SEMA with a higher QALY and lower cost. Figure 5 shows that there was a 68.7% probability that DULA was dominant to e-r EXEN and a 31.3% probability that e-r EXEN was not more cost-effective than DULA. Therefore, the base-case results were robust to the 1-w SA, scenario analysis, and PSA.

### 3.3 Binary Search for Exploring a Suitable Price Reduction for sc. SEMA and e-r EXEN

As shown in previous results, DULA appears to be the most cost-effective therapy among sc. SEMA, DULA, and e-r EXEN in treating T2D patients receiving metformin-based background therapy. Therefore, a spectrum of assumptions were made to explore a suitable price reduction for sc. SEMA and e-r EXEN using binary search, taking the cost of DULA as a reference. The detailed data on the hypothesis of annual costs and outcomes of cost-utility analysis for sc. SEMA or e-r EXEN are shown in Supplementary Table S1 [see additional file] and Supplementary Table S2 [see additional file]. When the annual cost of sc. SEMA was set at $1226.23, the ICUR of sc. SEMA vs. DULA was $31510.58/QALY, nearly approaching λ. That is, if there was a 57.67% reduction in sc. SEMA cost, sc. SEMA would become comparatively the same as DULA with regard to cost-effectiveness. Similarly, when the annual cost of e-r EXEN was set at $1067.41, the ICUR of DULA vs. e-r EXEN was $31510.50/QALY, nearly approaching λ. This means that if there was a 70.34% reduction in e-r EXEN cost, e-r EXEN would just reach comparatively the same cost-effectiveness as DULA.

## 4 Discussion

T2D causes progressive destruction of health, with most patients eventually experiencing various complications and even death. The proportion of patients who had at least one microvascular or macrovascular complication was 31.5% or 16.6%, respectively, after three years of follow-up in the longitudinal global DISCOVER study (Arnold et al., 2021). The study also pointed out that a higher HbA1c level and smoking habit made considerable contributions to the high risk of both microvascular and macrovascular complications (Arnold et al., 2021). Diabetic patients mainly suffer from morbidity and death due to cardiovascular diseases. The expenditure of T2D patients with complications was 3.46 times that of those without complications (Wu et al., 2019). GLP-1 RAs represent a novel class of treatments that offer multifaceted benefits, including glycemic control and protection of kidneys and heart (Buse et al., 2020; Chinese Society of Endocrinology, Chinese Diabetes Society, 2020). In particular, certain GLP-1 RAs have long-term benefits in protecting the heart and kidneys. For instance, the international guidelines recommend liraglutide, semaglutide, and dulaglutide to patients at high risk for cardiovascular events due to the long-term benefits of these GLP-1 RAs (Nauck and Quast, 2021). Furthermore, GLP-1 RA is the only drug class that reduces the risk of stroke (Lin et al., 2021). At present, GLP-1 RAs weekly formulations marketed in China include sc. SEMA, DULA, and e-r EXEN. Among them, DULA is currently included in the drug list of the national medical insurance and reimbursed only in patients with a BMI >25 kg/m^2^ whose diabetes is not controlled by metformin or insulin.

The increasing prevalence and socioeconomic burden make T2D an urgent public health matter. It is necessary to carry out pharmacoeconomic studies on diabetes therapies to alleviate the personal or social burden. Recently, cost-effectiveness analyses of GLP-1 RAs have been launched in many countries (Chuang et al., 2016; Ericsson and Fridhammar, 2019; Johansen et al., 2019; Vidal et al., 2020). However, different conclusions have been drawn in different countries due to different national conditions. The National Health and Nutrition Examination Survey pointed out that most T2D patients failed to reach treatment targets recommended by guidelines in the USA. At low and high doses, sc. SEMA was superior to DULA in terms of HbA1c reduction and body weight reduction in T2D patients in the SUSTAIN 7 trial (Pratley et al., 2018). A short-term economic estimation derived from the SUSTAIN 7 trial showed that most T2D patients treated with sc. SEMA achieved HbA1c targets and spent less than those treated with DULA from the perspective of a private health care payer in the USA for one year (Wilkinson et al., 2018). From the Slovakian perspective, sc. SEMA has improvements in QALYs with saving costs compared to DULA (Malkin et al., 2019). DULA was a dominant option with higher benefits (lifetime QALYs: 9.804 vs. 9.757) and lower costs (lifetime costs €41,562 vs. €43,021) than e-r EXEN for T2D payers in France (Basson et al., 2018). Compared with DULA and e-r EXEN for the treatment of patients with T2D, sc. SEMA could provide a cost-effective alternative to other GLP-1 RA therapies available with higher QALYs and lower costs in the Danish setting and UK setting (Gæde et al., 2019; Johansen et al., 2020). However, there was no previous economic evidence for sc. SEMA, DULA, and e-r EXEN for T2D patients with inadequate control who were on metformin-based background therapy from a Chinese perspective.

This study is the first to compare the long-term outcomes of three once-weekly GLP-1 RAs from a Chinese perspective using UKPDS OM2. Our present long-term cost-utility analysis found that, in China, DULA appears to be the most pharmacoeconomic strategy among DULA, sc. SEMA, and e-r EXEN in treating T2D patients with inadequate control by metformin-based background therapy. The base-case analysis was robust to 1-w SA, scenario analysis, and PSA. The clinical efficacy data were sourced from a meta-analysis that included 302 trials with 231,335 patients. In terms of the short-term efficacy from the meta-analysis, the changes in HbA1c levels in patients receiving metformin-based background therapy for DULA, sc. SEMA, and e-r EXEN were −0.89%, −1.33%, and −0.8%, respectively. Diabetes is chronic and progressive; therefore, long-term outcomes should be valued. In terms of the long-term outcomes from the cost-utility analysis, DULA, sc. SEMA, and e-r EXEN yielded 9.60, 9.63, and 9.59 QALYs, respectively. SEMA had the best clinical effect among the three, which was in line with the short-term results. Nevertheless, sc. SEMA was the newest GLP-1 RA listed on the Chinese market, and the unit price of sc. SEMA was also the highest among the three. After long-term estimation, sc. SEMA was not cost-effective, even though it had a clinical advantage. Therefore, it is imperative to lower the price of sc. SEMA appropriately. E-r EXEN brings little benefit but costs more to diabetes patients in the short- or long-term. Hence, cutting the price of e-r EXEN is also necessary. DULA entered the Chinese market in 2019 and was added to the drug list of the national medical insurance in 2020. In the binary search, several assumptions for the annual costs of sc. SEMA and e-r EXEN were input into UKPDS OM2 to identify a reasonable price for sc. SEMA and e-r EXEN, with DULA as a reference. Ultimately, sc. SEMA and e-r EXEN appear to be cost-effective when the annual cost decreases by 57.67% and 70.34%, respectively. This study offers a framework for estimating the long-term cost-effectiveness of the three GLP-1 RAs and exploring the reasonable reduction for sc. SEMA and e-r EXEN in the China marketplace.

As with all pharmacoeconomic analyses, the limitations in the modeling approach must be considered when translating the findings to clinical practice. First, similar to most economic evaluations for chronic diseases, the current research applied short-term clinical effectiveness to obtain long-term health outcomes. Even though this procedure is extensively projected in pharmacoeconomic studies and recommended by guidelines, it is still connected with considerable uncertainty. However, with a lack of real-world data over a long-term period, model simulation would be the best approach for predicting multidimensional effects in the future. Second, the equations in the UKPDS OM2 were deduced from the UKPDS 82 study, in which the patient cohort comprised Caucasian, Black, and Asian individuals. Hence, it is necessary to be cautious when extrapolating the model results to other populations. In addition, the clinical data from the meta-analysis were sourced from international randomized clinical trials, due to the lack of large-scale head-to-head clinical trials in China. These uncertainties were measured by sensitivity analysis. Ultimately, the procedure of drug pricing is complicated, and there are multiple factors that have to be considered. Therefore, economic suggestions serve only as a reference in the process of government adjustments to prices, and other diversified factors should also be comprehensively considered. In a word, caution should be taken when conclusions are applied to real-world market decisions.

## 5 Conclusion

From the Chinese health care provider’s perspective, DULA appears to be the most cost-effective option among sc. SEMA, DULA, and e-r EXEN for the treatment of T2D patients receiving metformin-based background therapy. A 57.67% or 70.34% reduction in cost would allow sc. SEMA or e-r EXEN, respectively, to become as cost-effective as DULA in China.

## Data Availability

The original contributions presented in the study are included in the article/Supplementary Material, further inquiries can be directed to the corresponding author.

## Abbreviations

1-w SA, one-way sensitivity analysis; CHF, congestive heart failure; CPI, consumer price index; DPP-4i, dipeptidyl peptidase-4 inhibitors; DULA, dulaglutide; e-r EXEN, extended-release exenatide; GDP, gross domestic product; GLP-1 RAs, glucagon-like peptide-1 receptor agonists; ICUR, incremental cost-utility ratio; IDF, International Diabetes Federation; IHD, ischemic heart disease; INMB, incremental net monetary benefit; LE, life expectancy; MI, myocardial infarction; PSA, probabilistic sensitivity analysis; QALYs, quality-adjusted life years; sc. SEMA, subcutaneous semaglutide; SGLT-2i, sodium–glucose cotransporter-2 inhibitors; T2D, type 2 diabetes; UKPDS OM2, United Kingdom Prospective Diabetes Study Outcomes Model (version 2); WHO, World Health Organization.
