# Supplementary material for: Cost-Utility Analysis of Once-Weekly Semaglutide, Dulaglutide, and Exenatide for Type 2 Diabetes Patients Receiving Metformin-Based Background Therapy in China
Source: Front Pharmacol. 2022 Feb 18;13:831364. doi: 10.3389/fphar.2022.831364 (PMC8894868; doi:10.3389/fphar.2022.831364)
Supplement: Supplementary file 1 [file Table1.DOCX]

**Supplementary file 1**


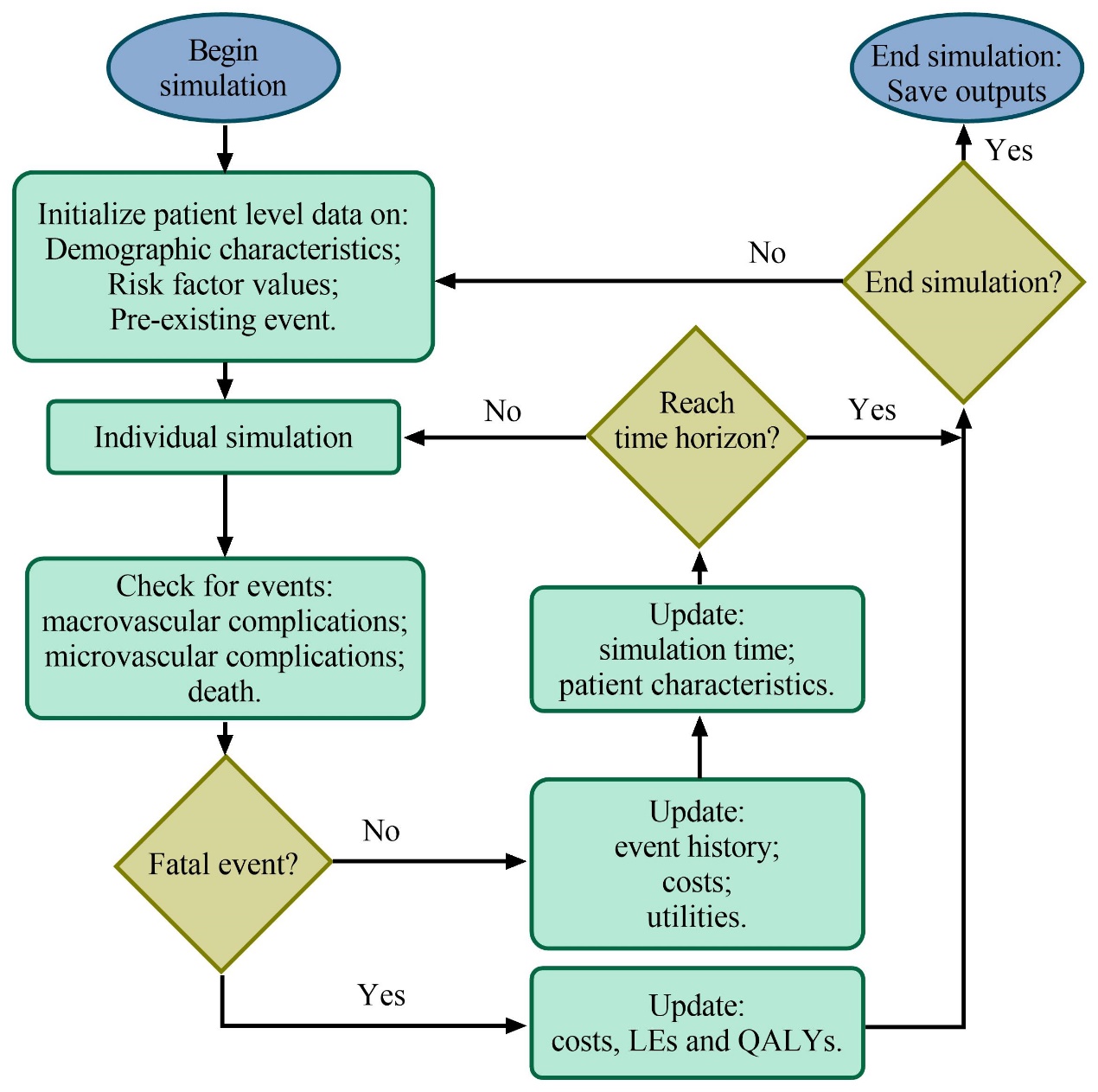


Figure S1 Diagram of the UKPDS OM2 model structure.

**Supplementary file 2**

Table S1 Outputs for cost-utility analysis testing for a suit price for sc. SEMA

| NO. | Code | Group | ∆QALY | ∆cost | ICUR | relationship with λ |
| --- | --- | --- | --- | --- | --- | --- |
| 1 | A | Baseline = $3,897.12 | | | | |
|  |  | SEMA | 0.0346478 | 17081.2 | 492994.7 | > |
|  |  | DULA |  |  |  |  |
| 2 | B | A*1/2 = $1,948.56 | | | | |
|  |  | SEMA | 0.0346478 | 5416.06 | 156317.4 | > |
|  |  | DULA |  |  |  |  |
| 3 | C | B*1/2 = $974.28 | | | | |
|  |  | SEMA | 0.0346478 | -416.51 | -12021.2 | < |
|  |  | DULA |  |  |  |  |
| 4 | D | (B+C)*1/2 = $1,461.42 | | | | |
|  |  | SEMA | 0.0346478 | 2499.775 | 72148.09 | > |
|  |  | DULA |  |  |  |  |
| 5 | E | (C+D)*1/2 = $1,217.85 | | | | |
|  |  | SEMA | 0.0346478 | 1041.633 | 30063.43 | < |
|  |  | DULA |  |  |  |  |
| 6 | F | (D+E)*1/2 = $1,339.64 | | | | |
|  |  | SEMA | 0.0346478 | 1770.704 | 51105.76 | > |
|  |  | DULA |  |  |  |  |
| 7 | G | (E+F)*1/2 = $1,278.74 | | | | |
|  |  | SEMA | 0.0346478 | 1406.168 | 40584.6 | > |
|  |  | DULA |  |  |  |  |
| 8 | H | (E+G)*1/2 = $1,248.30 | | | | |
|  |  | SEMA | 0.0346478 | 1223.901 | 35324.01 | > |
|  |  | DULA |  |  |  |  |
| 9 | I | (E+H)*1/2 = $1,233.07 | | | | |
|  |  | SEMA | 0.0346478 | 1132.767 | 32693.72 | > |
|  |  | DULA |  |  |  |  |
| 10 | J | (E+J)*1/2 = $1,225.46 | | | | |
|  |  | SEMA | 0.0346478 | 1087.2 | 31378.58 | < |
|  |  | DULA |  |  |  |  |
| 11 | K | (I+J)*1/2 = $1,229.27 | | | | |
|  |  | SEMA | 0.0346478 | 1109.983 | 32036.15 | > |
|  |  | DULA |  |  |  |  |
| 12 | L | (J+K)*1/2 = $1,227.36 | | | | |
|  |  | SEMA | 0.0346478 | 1098.591 | 31707.36 | > |
|  |  | DULA |  |  |  |  |
| 13 | M | (J+L)*1/2 = $1,226.41 | | | | |
|  |  | SEMA | 0.0346478 | 1092.896 | 31542.97 | > |
|  |  | DULA |  |  |  |  |
| 14 | N | (J+M)*1/2 = $1,225.94 | | | | |
|  |  | SEMA | 0.0346478 | 1090.048 | 31460.77 | < |
|  |  | DULA |  |  |  |  |
| 15 | O | (M+N)*1/2 = $1,226.18 | | | | |
|  |  | SEMA | 0.0346478 | 1091.472 | 31501.87 | < |
|  |  | DULA |  |  |  |  |
| 16 | P | (M+O)*1/2 = $1,226.29 | | | | |
|  |  | SEMA | 0.0346478 | 1092.184 | 31522.42 | > |
|  |  | DULA |  |  |  |  |
| 17 | Q | (O+P)*1/2 = $1,226.23 | | | | |
|  |  | SEMA | 0.0346478 | 1091.828 | 31512.14 | > |
|  |  | DULA |  |  |  |  |
| 18 | R | (O+Q)*1/2 = $1,226.21 | | | | |
|  |  | SEMA | 0.0346478 | 1091.65 | 31507.01 | < |
|  |  | DULA |  |  |  |  |
| 19 | S | (Q+R)*1/2 = $1,226.22 | | | | |
|  |  | SEMA | 0.0346478 | 1091.739 | 31509.58 | < |
|  |  | DULA |  |  |  |  |
| 20 | T | (Q+S)*1/2 = $1,226.23 | | | | |
|  |  | SEMA | 0.0346478 | 1091.783 | 31510.86 | > |
|  |  | DULA |  |  |  |  |
| 21 | U | (S+T)*1/2 = $1,226.22 | | | | |
|  |  | SEMA | 0.0346478 | 1091.761 | 31510.22 | < |
|  |  | DULA |  |  |  |  |
| 22 | V | (T+U)*1/2 = $1,226.23 | | | | |
|  |  | SEMA | 0.0346478 | 1091.772 | 31510.54 | < |
|  |  | DULA |  |  |  |  |
| 23 | W | (T+V)*1/2 = $1,226.23 | | | | |
|  |  | SEMA | 0.0346478 | 1091.778 | 31510.7 | > |
|  |  | DULA |  |  |  |  |
| 24 | X | (V+W)*1/2 = $1,226.23 | | | | |
|  |  | SEMA | 0.0346478 | 1091.775 | 31510.62 | > |
|  |  | DULA |  |  |  |  |
| 25 | Y | (V+X)*1/2 = $1,226.23 | | | | |
|  |  | SEMA | 0.0346478 | 1091.773 | 31510.58 | ≈ |
|  |  | DULA |  |  |  |  |

Table S2 Outputs for cost-utility analysis testing for a suit price for e-r EXEN

| NO. | Code | Group | ∆QALY | ∆cost | ICUR | relationship with λ |
| --- | --- | --- | --- | --- | --- | --- |
| 1 | A | Baseline = $3,599.38 | | | | |
|  |  | DULA | 0.0073405 | -15333.5 | Dominant | > |
|  |  | EXEN |  |  |  |  |
| 2 | B | A/2 = $1,799.69 | | | | |
|  |  | DULA | 0.0073405 | -4622.72 | Dominant | > |
|  |  | EXEN |  |  |  |  |
| 3 | C | B/2 = $899.85 | | | | |
|  |  | DULA | 0.0073405 | 798.3208 | 108755.2 | > |
|  |  | EXEN |  |  |  |  |
| 4 | D | (B+C)*1/2 = $1,349.77 | | | | |
|  |  | DULA | 0.0073405 | -1912.2 | -260499 | < |
|  |  | EXEN |  |  |  |  |
| 5 | E | (C+D)*1/2 = $1,124.81 | | | | |
|  |  | DULA | 0.0073405 | -556.94 | -75872 | < |
|  |  | EXEN |  |  |  |  |
| 6 | F | (C+E)*1/2 = $1,012.33 | | | | |
|  |  | DULA | 0.0073405 | 120.6902 | 16441.62 | < |
|  |  | EXEN |  |  |  |  |
| 7 | G | (C+F)*1/2 = $1,181.05 | | | | |
|  |  | DULA | 0.0073405 | 459.5055 | 62598.42 | > |
|  |  | EXEN |  |  |  |  |
| 8 | H | (F+G)*1/2 = $1,096.69 | | | | |
|  |  | DULA | 0.0073405 | 290.0978 | 39520.02 | > |
|  |  | EXEN |  |  |  |  |
| 9 | I | (F+H)*1/2 = $1,054.51 | | | | |
|  |  | DULA | 0.0073405 | 205.394 | 27980.82 | < |
|  |  | EXEN |  |  |  |  |
| 10 | J | (H+I)*1/2 = $1,075.60 | | | | |
|  |  | DULA | 0.0073405 | 247.7459 | 33750.42 | > |
|  |  | EXEN |  |  |  |  |
| 11 | K | (I+J)*1/2 = $1,065.05 | | | | |
|  |  | DULA | 0.0073405 | 226.57 | 30865.62 | < |
|  |  | EXEN |  |  |  |  |
| 12 | L | (J+K)*1/2 = $1,070.32 | | | | |
|  |  | DULA | 0.0073405 | 237.158 | 32308.02 | > |
|  |  | EXEN |  |  |  |  |
| 13 | M | (K+L)*1/2 = $1,067.69 | | | | |
|  |  | DULA | 0.0073405 | 231.864 | 31586.82 | > |
|  |  | EXEN |  |  |  |  |
| 14 | N | (K+M)*1/2 = $1,066.37 | | | | |
|  |  | DULA | 0.0073405 | 229.217 | 31226.22 | < |
|  |  | EXEN |  |  |  |  |
| 15 | O | (M+N)*1/2 = $1,067.03 | | | | |
|  |  | DULA | 0.0073405 | 230.5405 | 31406.52 | < |
|  |  | EXEN |  |  |  |  |
| 16 | P | (M+O)*1/2 = $1,067.36 | | | | |
|  |  | DULA | 0.0073405 | 231.2022 | 31496.67 | < |
|  |  | EXEN |  |  |  |  |
| 17 | Q | (M+P)*1/2 = $1,067.52 | | | | |
|  |  | DULA | 0.0073405 | 231.5331 | 31541.75 | > |
|  |  | EXEN |  |  |  |  |
| 18 | R | (P+Q)*1/2 = $1,067.44 | | | | |
|  |  | DULA | 0.0073405 | 231.3677 | 31519.21 | > |
|  |  | EXEN |  |  |  |  |
| 19 | S | (P+R)*1/2 = $1,067.40 | | | | |
|  |  | DULA | 0.0073405 | 231.2849 | 31507.94 | < |
|  |  | EXEN |  |  |  |  |
| 20 | T | (R+S)*1/2 = $1,067.42 | | | | |
|  |  | DULA | 0.0073405 | 231.3263 | 31513.58 | > |
|  |  | EXEN |  |  |  |  |
| 21 | U | (S+T)*1/2 = $1,067.41 | | | | |
|  |  | DULA | 0.0073405 | 231.3056 | 31510.76 | > |
|  |  | EXEN |  |  |  |  |
| 22 | V | (S+U)*1/2 = $1,067.40 | | | | |
|  |  | DULA | 0.0073405 | 231.2953 | 31509.35 | < |
|  |  | EXEN |  |  |  |  |
| 23 | W | (U+V)*1/2 = $1,067.41 | | | | |
|  |  | DULA | 0.0073405 | 231.3004 | 31510.06 | < |
|  |  | EXEN |  |  |  |  |
| 24 | X | (U+W)*1/2 = $1,067.41 | | | | |
|  |  | DULA | 0.0073405 | 231.303 | 31510.41 | < |
|  |  | EXEN |  |  |  |  |
| 25 | Y | (U+X)*1/2 = $1,067.41 | | | | |
|  |  | DULA | 0.0073405 | 231.3043 | 31510.58 | > |
|  |  | EXEN |  |  |  |  |
| 26 | Z | (X+Y)*1/2 = $1,067.41 | | | | |
|  |  | DULA | 0.0073405 | 231.3037 | 31510.5 | < |
|  |  | EXEN |  |  |  |  |
